# Laser capture microdissection to study Bacillus cereus iron homeostasis gene expression during Galleria mellonella in vivo gut colonization

**DOI:** 10.1080/21505594.2021.1959790

**Published:** 2021-08-10

**Authors:** Laurent Consentino, Agnès Rejasse, Nicolas Crapart, Claudia Bevilacqua, Christina Nielsen-LeRoux

**Affiliations:** a Université Paris-Saclay, INRAE, AgroParisTech, Micalis Institute, Jouy-en-Josas, France; b Université Paris Saclay, INRAE, AgroParisTech, UMR GABI, Abridge, Jouy En Josas, France; c Exilone, Elancourt, France

**Keywords:** Laser-capture microdissection, *bacillus cereus*, *galleria mellonella*, iron, intestinal infection, *in situ* qPCR, gene expression, histology, colonization, insect model

## Abstract

*Bacillus cereus* is a Gram-positive opportunistic pathogen closely related to the entomopathogen, *Bacillus thuringiensis*, both of which are involved in intestinal infections. Iron is an essential micronutrient for full growth and virulence of pathogens during infection. However, little is known about iron homeostasis during gut infection. Therefore, we aimed to assess the expression of *B. cereus *genes related to bacterial iron homeostasis, virulence and oxidative stress. The hypothesis is that the expression of such genes would vary between early and later stage colonization in correlation to gut cell damage. To perform the study, a germ-free *Galleria mellonella* model was set up in order to adapt the use of Laser-capture microdissection (LCM), to select precise areas in the gut lumen from frozen whole larval cryo-sections. Analyses were performed from alive larvae and the expression of targeted genes was assessed byspecific pre-amplification of mRNA followed by quantitative PCR. Firstly, the results reinforce the reliability of LCM, despite a low amount of bacterial RNA recovered. Secondly, bacterial genes involved in iron homeostasis are expressed in the lumen at both 3 and 16 hours post force-feeding. Thirdly, iron gene expression is slightly modulated during gut infection, and lastly, the mRNA of *G. mellonella* encoding for ferritin and transferrin iron storage and transport are recovered too. Therefore, iron homeostasis should play a role in *B. cereus* gut colonization. Furthermore, we demonstrate for the first time the value of using LCM for specific *in situ* gene expression analysis of extracellular bacteria in a whole animal.

## INTRODUCTION

Iron is an essential micronutrient for the majority of living organisms. It is involved in several vital processes such as DNA synthesis, respiration, and several other biochemical reactions. However, iron can trigger Fenton reactions, which generates reactive oxygen species a threat for cell membrane integrity, nucleic acids, and proteins [1,2]. Therefore, iron homeostasis is tightly regulated in living organisms. In man and other mammals, the majority of iron is bound to heme in hemoproteins, or bound to transport proteins like transferrin or stored in ferritins, which can reserve thousands of iron atoms [3,4].

To overcome this lack of free iron, pathogens are equipped with mechanisms capable of grabbing iron from the iron-rich host molecules [5,6] or hijacking host iron transport molecules [7]. In this study, we target the opportunist food-borne pathogen *Bacillus cereus sensu stricto*, a Gram-positive, spore-forming bacteria causing emetic or diarrheal symptoms in humans [8,9]. The *Bacillus cereus sensu stricto* is tightly related to the human pathogen *Bacillus anthracis* and the entomopathogen *Bacillus thuringiensis* [10–13]. *B. cereus* possesses different strategies to scavenge sequestered iron during host infection. This can be done through direct interactions using surface proteins [14–16], or indirect interactions using both surface proteins to bind the iron source and secreted siderophores, which are small molecules with high affinity for free Fe^3+^ [17]. The siderophore bacillibactin, encoded by genes of the *entA-dhbBCF* cluster [18], has been shown to have a major impact on bacterial survival in a low iron environment. For example, the *B. cereus* ATCC 14579 bacillibactin mutant is strongly affected *in vitro* in iron deprived conditions, and in virulence, following injection into the hemocoel of the insect larvae *Galleria mellonella* [19]. The *feuABCD/yuil* operon codes in *B. cereus* for an ABC carrier type protein supposed to allow the transport of the bacillibactin/Fe3 + complex into the cell, with the gene *feuA* encoding for the specific siderophore binding protein [20]. Like for several other bacteria, the processes involving iron homeostasis-related gene expression in *B. cereus*, are also under the control of the repressor Fur (Ferric Uptake Regulator), which modulates indirectly pathogenicity during host infection [21–23]. Furthermore, *in vitro* transcriptomic studies were performed in *B. cereus* in iron-depleted conditions highlighting genes which are involved in iron homeostasis [24].

However, little is known of the role played by genes related to iron homeostasis during gut infection and colonization in pathogenic Gram-positive bacteria. In the Gram-negative *Escherichia coli*, it was found that the catecholate type siderophores and their associated transport systems have a role in the colonization of the mouse urinary tract and gut [25,26]. In addition, genes involved in iron uptake confer fitness advantages to *Salmonella enterica* during growth and colonization in the mouse intestine [27]. Likewise, gut microbiota and probiotic bacteria are reported to be involved in iron uptake in the gut, thereby preventing sustainable colonization of several bacterial species like *Salmonella* [28,29]. Strategies targeting siderophore acquisition of enteric pathogens have been tested as new therapeutic and preventive strategies [30].

*G. mellonella* is used as a promising and low-cost infection model since it harbors a relatively complex innate immune system similar to mammals [31,32]. The larval stage has been used to analyze Gram-negative (*e.g. Pseudomonas aeruginosa, Escherichia coli*) or Gram-positive bacteria (*e.g. Staphylococcus aureus, Enterococcus faecalis, Listeria monocytogenes*), allowing identification of virulence factors triggering mammalians infections and antimicrobial drugs testing following injection into the hemocoel [33,34]. *G. mellonella* was also proved to be an appropriate model for *B. cereus, B. thuringiensis, Pseudomonas entomophila,* and *Listeria monocytogene*s oral infection studies [35–37] and for a *B. cereus* IVET (*in vivo* expression technology) promoter trap screening [38]. The latter approach identified IlsA a unique surface protein involved in iron acquisition [14,15] and revealed expression of other iron regulated factors during infection [38]. To decipher interactions between microorganisms and the host during infection, we have chosen a challenging approach based on laser-capture microdissection (LCM) [39,40]. This technology allows the specific isolation of a target region without impact on the gene expression profile and has been used in different organisms and ecological niches [41,42]. Originally, LCM was used in cancer research [43–45] and then in broad-range disciplines in life sciences using mostly mammals as model. But, so far, only few examples are related to microbiology, for instance, to obtain information on the microbiota species spatial organization in the gut [46–48], and host responses comparing conventional versus germ-free enterocytes [49]. Reports on gene expression of an intracellular bacterial pathogen [50] or plant-microorganisms interactions [51,52] are rare.

In our study, we have developed a method of breeding germ-free *Galleria mellonella* larvae to avoid measuring commensal microbiota gene expression. We aimed to explore different *B. cereus* genes, with focus on those reported or supposed as being involved in iron homeostasis. We also selected a few specific virulence factors previously shown to be involved in gut infection along with factors involved in oxidative stress. We wanted to explore the expression of these genes in the insect gut environment as they may play a central role in bacterial settlement and indirectly or directly in virulence in the host gut. LCM was used for selection of bacteria from the intestine lumen at early and late gut colonization from whole snap frozen larvae. The main objective of our study was to analyze bacterial gene expression, but the expression of a few iron and immunity-related genes of the *G. mellonella* larvae were also analyzed. The expression of the -iron storage and transport proteins ferritin and transferrin, which have similar function in insects and mammals, were measured [B. Y. 53–55]. To our knowledge, the use of LCM for a precise gene expression analysis in an extracellular pathogen during gut colonization has never been reported before. Therefore, the approach is innovative and should be relevant for other host pathogen *in vivo* and *in situ* gene expression analyses.

## Material and Methods:

### B. cereus *growth conditions for* Galleria mellonella *infection*

*Bacillus cereus* ATCC 14579 (laboratory stock) was used in this study. For infection and colonization, the strain was cultured in 10 mL volume of LB (Luria-Bertani) broth, with vigorous shaking (190 rpm) at 37°C. At OD_600 nm_ = 1, bacteria were harvested by centrifugation (8000 rpm) and washed twice with sterile phosphate saline buffer (PBS) 1X (filtered on a 0.22 µm membrane, GP Millipore Express® PLUS Membrane).

### B. cereus *growth and qPCR for* in vitro *conditions*

The *B. cereus* ATCC 14,579 strain, as control for RT qPCR analyses, was cultured in 10 mL LB or 10 mL LB with an iron chelator, the 2.2’-Bipyridyl (DIP) at 200 µM, with vigorous shaking (190 rpm) at 37°C and collected at their respective mid-log growth phase. Each condition (LB and LB + DIP 200 µM) was performed in three biological replicates. Cultures were then centrifuged at 10,000 rpm during 5 minutes. The supernatants were discarded and pellets were stored at – 80°C before total RNA extraction. Extraction was performed as described by 56. The qPCR was performed on QuantStudio™ 12 K Flex [Applied Biosystems, Thermo Fisher Scientific] as described by 57. Data analysis was conducted by the comparative threshold cycle method (ΔΔCt) with the Thermo Fischer Cloud using the statistical test Benjamini-Hochberg false discovery rate procedure.

### *Germ-free* Galleria mellonella *set up*

All the devices and material used for the preparation of germ-free *G. mellonella* were cleaned with 70% ethanol prior to a 10-minute UV light (254 nm) exposure in sterile conditions (Microbiological safety cabinet, MSC). Further manipulations including *G. mellonella* infections were performed within a sterile MSC. To generate germ-free larvae, UV-treated *G. mellonella* eggs were reared on sterile (Cobalt irradiated intensity 25 kGy (SAFE Company, Augy, France)) beeswax and pollen. Glass jars containing eggs and food were sealed (permitting aeriation with filtering cotton on the lid) and incubated at 27°C in a humidified incubator. Verification of bacterial germ-free (bacteria only) status was assessed by plating on 3 LB agar petri-dishes, with 300 µL suspension of a larvae homogenized in 1 ml sterile PBS (3 larvae/batch) incubated at 37°C for 24 h. In addition, extraction of DNA from the dissected digestive tract was performed for further verification. DNA quantity and quality were assessed (NanoDrop 2000, Thermo Scientific) and PCR amplification of 16S rRNA gene using V3 and V4 primers for bacterial DNA detection was conducted. No amplification of bacterial DNA was found in larvae from the three batches, while amplification was obtained with DNA from normal reared larvae.

### *Gut colonization estimation and mortality assays in* Galleria mellonella

Last instar axenic reared larvae (250–300 mg) were force-fed as previously described [58] with a mixture of mid log phase (OD 600 = 1, LB medium) vegetative *B. cereus* ATCC 14,579 strain bacteria suspended in trypsin activated Cry1C toxin. This is the so-called synergy model, in which no infection or mortality occurs with the force-feeding of the toxin or bacteria alone, while the association of bacteria and the toxin are able to infect the insect resulting in mortality [37]. Forty larvae were force-fed for each of the three independent experiments. Larval mortality/survival was scored at 3, 16, and 24 hours post force-feeding. To estimate colonization, triplicates of two larvae were analyzed, for a total of 6 larvae per time point or 18 larvae per experiment. The remaining larvae were used to measure the survival rate. The Bacteria used for force-feeding were harvested by centrifugation and the pellet suspended in Cry1C toxin (0.3 mg/mL) at 5 × 10^8^ colony forming unit (CFU)/mL resulting in a 10 µl dose of 5 × 10^6^ CFU and 3 µg Cry1C toxin/larva. Larvae were incubated at 37°C without food. Bacterial colonization was scored by counting *B. cereus* CFUs in the living insects at T0 (just after) and 3 hours and 16 hours post force-feeding, each sample of 2 larvae were crushed and homogenized with UltraTurax tissue cutter in a final volume of 10 mL sterile water; dilutions were immediately plated onto LB agar. For each sample, three technical replicates were performed to score CFU.

### *Preparation of frozen* Galleria *for Laser capture microdissection*

As described, the axenic last instar larvae (250–300 mg) were force-fed [58] with the same *B. cereus* ATCC 14579 mid log phase centrifuged bacteria from LB medium mixed with Cry1C, as in the above colonization experiment. Larvae were first incubated at 37°C and then frozen in isopentane (Sigma-Aldrich) in a Snapfrost equipment (Excilone, France) at 3 and 16 hours post forcefeeding. Control larvae without bacteria were force-fed with 10 µL of either Cry1C toxin (0.3 mg/mL) or sterile PBS, in order to investigate the effect on the peritrophic matrix and gut cells with the toxin alone. No test was run with the bacteria alone since in that case the bacteria would just transit inside the peritrophic matrix space (in the food bolus) to be excreted with the feces after 2 hours [59].

### Tissues Preparation for LCM

The cryo-section process was performed in a cryostat (FSE Shandon, ThermoFisher, France) at −20°C and in an RNA-free environment (material treated with RNAzap and 70% ethanol). The frozen infected larvae were stored at −80°C until LCM was performed. For each biological replicate and to obtain enough material for downstream analyses, the second quarter of five larvae were cut out and 10 cross-sections per quarter were collected on Arcturus PEN membrane RNAse-free glass slides (Excilone, France). Approximately 3 to 5 sections were gathered on each slide, and fixed immediately in 75% cold ethanol in the cryostat before further processing. The larval cross-sections (16 µm thick) were then dehydrated and colored by the following protocol [60,61]: 50% ethanol for 20 seconds, homemade ethanolic solution of 1% cresyl violet for 20–30 seconds, 50% ethanol for 20 seconds, 75% ethanol for 30 seconds, 95% ethanol for 40 seconds (twice), 100% ethanol for 1 min (twice), xylene for 5 min (twice). To locate the targeted structures, sections were analyzed under the optical microscope using the XT® Arcturus Technologies microdissection system (Excilone, France).

### Laser Capture Microdissection

The LCM process was carried out using the XT® Arcturus Technologies microdissection system and software. Capture was performed using Arcturus CapSure® LCM Macro Caps (Excilone, France). Bacteria present inside the insect gut were identified using the 20X objectives. PEN Membrane Slides allowed the use of standard IR and UV lasers. UV laser was used to cut around the lumen, avoiding epithelial cells, and IR laser was used to capture and stick the samples on the macro caps without contaminating the sample with non-target tissue. Efficiency of microdissection was evaluated by examining the samples after lifting off the CapSure® macro caps. To obtain enough material for downstream analysis, 5 to 10 caps *per* biological replicate were stored at −80°C.

### RNA extraction from LCM samples

For each condition (3 or 16 hours) and biological replicates (three biological replicates overall), the samples sticking over the caps were gently collected using a RNase-free device to remove the film covering the caps, and pooled in the same RNase-free tube. Total RNA was extracted using the Picopure™ RNA Isolation kit (Excilone, France). The protocol recommended by the manufacturer was modified to adapt with Gram-positive bacteria characteristics. To reach an optimal total RNA extraction, the following steps were performed:

i) A volume corresponding to 50 µL of lysozyme solution (10 mM Tris-HCl pH = 8/0.1 mM ethylenediaminetetraacetic acid (EDTA)/1 mg lysozyme) by cap was added in each tube and thoroughly mixed ; ii) Addition of 0.5 µL of a 10% Sodium Dodecyl Sulfate (SDS) solution to the lysate and well mixed; iii) Incubation 5 minutes at room temperature; iv) Addition of 150 µL of Extraction Buffer (provided by manufacturer); v) The lysate was gently aspired 5 times through a RNAse free needle and syringe; vi) Addition of 70% ethanol.

The protocol was ended following the manufacturer procedure. The quality and quantity of the RNA was assessed using an Agilent 2100 Bioanalyzer (Agilent Technologies, Palo Alto, CA) with an RNA6000 Pico Lab Chip and analyzed by the RNA Integrity Number (RIN) algorithm [62].

### Targeted genes for Quantitative PCR

For this study, we targeted genes involved in *B. cereus* iron homeostasis along with other genes implicated in different bacterial processes like tissue damage, oxidative stress, etc. Reference housekeeping genes *rpoB, tpi* and *purH* were tested for accurate normalization of data (Table 1).

**Table 1.** *Bacillus cereus* ATCC 14579 genes targeted in the qPCR analysis classified by function

**Locus tag**	Name	Primers (up: forward; down: reverse)5’ to 3’	Function (annotated)
**Reference genes**			
BC0122	*rpoB*	CGGCAGCGACAGCTTGTATT	DNA-directed RNA polymerase subunit beta
		GCATGTTCGCTCCCATAAGTG	
BC5137	*tpi*	GAACTGACTTACAAGTAGGTGCACAAA	Trio phosphate isomerase
		TCGCCAGTGAATGCACCAT	
BC0333	*purH*	GGTAGCGCACTTGCATCTGA	Bifunctional phosphoribosylaminoimidazolecarboxamide formyltransferase/IMP cyclohydrolase
		TCCTGCTTTTGCTGCTTCTTC	
**Iron homeostasis**			
BC1331	*ilsA*	TGGCCATTGGGCGAAA	Iron regulated leucine-rich protein
		CCCCGTCGCCTTTTGATAT	
BC2302	*entA*	CGGCAAATACGCCGAGAAT	3,3-dihydro-3,3-dihydroxybenzoate dehydrogenase (gene from bacillibactin operon)
		ACATCGTCGTTGCAGCCTTT	
BC3738	*feuA*	AGTGCCAGCAAAAGTAGATAAAATTG	Bacillibactin siderophore binding protein
		TTCCAAGTACTGCCGCATCTT	
BC5106	*fatB*	GGTTTTGCTGCGCCACAT	Petrobactin siderophore binding protein
		AGTCCAACCGAAAATTTCATACATT	
BC4528	*fpuA*	ATATGGATAAAGCATTCGCTGATG	Petrobactin siderophore binding protein
		CCATTGCGATGTTTTTATCTTTTAAG	
BC4549	*isdC*	AGTGCAAACTATGACCACCACTACA	Heme iron transport associated protein
		TCGCCACCTACAGCTTTTACATT	
BC0707	*feoB*	ACTTACCACGTGTCGCGTTTAA	Ferrous iron transport protein
		TGTGCGCCGGATCGTT	
BC0616	*fec*	GGCAAACTTGGCGATAAAGTAAA	Iron (III) dicitrate ABC transporter
		ACGAACATCACCAGGCATGAA	
BC1978-BC1983	*asb*	ACATGCAAACGACGCAACA	Petrobactin siderophore synthesis locus
		GCGTACGCTCTCCAGGTATAGC	
**Regulation of genes expression**			
BC4091	*fur*	ACACCGCAACGTGAAGCA	Ferric uptake regulation protein
		TCTGCGCTTAAATGATCTTCTTCAT	
BC5350	*plcR*	CGATTGAAATATCATGCCGAATT	Transcriptionnal activator PlcR
		AGGCATTCACCTCTTTGATAATATAGC	
BC3826	*codY*	CGTAGTTAGCCGTCGTGGTAAA	Transcriptional repressor CodY
		TGCGTTCGTTTTCGATTTGTT	
**Oxidative stress**			
BC5445	*sodA*	CTTGACGGTGAGGAACTCTCTGT	Superoxide dismutase
		CCCTTCTTGCAAAGGCGTATC	
BC1155	*catalase*	TGGTGCGATGCAAGTGAATC	Catalase enzyme
		CATGACGGCTCGGTTCGTA	
BC5044	*dps*	AGAAGGGATGGTCGAAGCAA	Ferritin-like iron-binding protein
		GCCTTTTTTCAGTTCGATTAGCA	
**Virulence factors**			
BC1809	*nheB*	AAGACTTTAATTACAGGGTTATTGGTTACA	Non hemolytic enterotoxin lytic L2 (PlcR Regulon)
		TCTGTTTGCCCCTCCTTAGC	
BC3523	*hlyII*	CTGGAAAAACCATCAAGTTACTC	Hemolysin II (Fur regulon)
		TCACCATTTACAAAGATACC	
BC0666	*inhA2*	AATTATGCGGGATCAGATAATGG	Immune inhibitor A precursor (PlcR regulon)
		GATGCACCCCAACCCAGTTA	

Primers were designed using Primer Express™ 3 software (Applied Biosystems), adapting the algorithm of primer research for SYBR Green quantitative PCR (qPCR). Primer efficiencies were tested on a cDNA pool obtained from *B. cereus* cultured in LB medium and LB with an iron chelator, the 2,2’-Bipyridyl (DIP), with vigorous shaking (190 rpm) at 37°C and stopped at OD_600nm_ = 1. Primer efficiencies were tested using standard curves with different concentrations of *B. cereus* cultures cDNA and melting curve. All primers had at least 85% of efficiency. Two *G. mellonella* host genes (Table 2) related to iron homeostasis and nutrition immune responses were analyzed as markers for host reaction to iron modulation during infection: the iron-transport protein transferrin, and the iron storage and transport molecule ferritin 32 kDa subunit present in *G. mellonella* gut cells [B. S. 63]. The chosen *G. mellonella* reference genes were the 18S rRNA and the elongation factor EF1 as described earlier [64].

**Table 2.** *Galleria mellonella* genes targeted in the qPCR analysis classified by function

**Locus tag**	Name	Primers (5’ to 3’)	Function (annotated)
**Reference genes**			
AF286298	*18S rRNA*	Fw : CACATCCAAGGAAGGCAG	Structural ribosomal RNA
		Rv : AGTGTACTCATTCCGATTACGA	
AF423811	*EF1*	Fw : AACCTCCTTACAGTGAATCC	Elongation factor 1-alpha (Ef-1a)
		Rv : ATGTTATCTCCGTGCCAG	
**Iron homeostasis**			
AY364430	Transferrin	Fw : CGTAGCAGTCATCAAGAAGG	Transferrin precursor
		Rv : CGCACTCACTAGAACTGG	
Gm_Ferr32k	Ferritin	Fw : TGCTTCCTCGCCGTGTCTG	*Galleria mellonella* 32 kDa ferritin
		Rw : TGCATCTCTGTGGCGACGTT	

### mRNA amplification and Quantitative PCR (qPCR)

Reverse transcription (RT) to obtain cDNA was performed using the TATAA Grandscript cDNA Synthesis Kit (TATAA Biocenter) according to the manufacturer protocol, to allow RT of low copy number targets, with a GeneAmp PCR System 2400 (PERKIN ELMER).

Before the qPCR, a pre-amplification (PreAmp) step was needed, as the amount of bacterial mRNA was too low in the collected samples to get a correct detection during the SYBR Green qPCR analysis. To perform this pre-amplification, we used the TATAA PreAmp GrandMaster® Mix (TATAA Biocenter). The first step was to prepare a primer pool in nuclease free water (Qiagen) of primers targeting the selected genes. Accordingly, with the manufacturer recommendations, each couple of primers in the primers pool had a final concentration of 50 nM. To test the efficiency of PreAmp, we used the same pool of *B. cereus* total RNA extracted. The PreAmp mix was then prepared as following for each biological replicate: 25 µL of TATAA PreAmp GrandMaster® Mix; 20 µL of primers pool (20 nM final); 5 µL of undiluted cDNA. The cycling protocol respects the manufacturer recommendations with 18 cycles in a GeneAmp PCR System 2400 (PERKIN ELMER). The PreAmp reliability was tested before the experiments, using two different concentrations of total RNA extracted from a *B. cereus* cells pool cultured in LB and in LB + DIP 0.2 mM. The concentrations were 50 pg and 0.05 pg of total RNA. RT and PreAmp were performed as described above, and control (cDNA without PreAmp) was analyzed too. The relative Ct gain after qPCR was then determined.

SYBR Green qPCR (SYBR qPCR) was performed following the instructions of TATAA Biocenter, using the QuantStudio™ 12 K Flex (Applied Biosystems by Thermo Fisher Scientific). For each sample, 2 µL of PreAmp cDNA was loaded with 10 µL of TATAA SYBR® GrandMaster® Mix (1X final), 1.2 µL of forward and reverse primer (300 nM each) and 2.6 µL of nuclease free water (provided with the kit). The three steps cycling protocol was as following: Pre-denaturation: 95°C, 30 seconds; Cycling: 95°C, 5 seconds followed by 60°C, 30 seconds; Data collection: 70°C, 10 seconds. Data were primarily analyzed and collected with the QuantStudio™ 12 K Flex Software v1.2.2 and further analyses were performed using the Thermo Fisher Connect™ cloud.

## Results

In this study, we wanted to analyze *B. cereus* gene expression while the bacteria were present in the intestinal lumen or colonizing the intestinal cells before larval mortality. For that purpose, we used the *G. mellonella* synergy model (developed in our team), for which no or low mortality is observed when *G. mellonella* is fed with Cry1C toxin or *B. cereus* alone, while a high mortality is reached when the bacteria are associated with the toxin [37]. The bacteria alone were not tested as it is known that the bacteria alone cannot infect the *G. mellonella* larva, as they are excreted rapidly along with the peritrophic matrix, when force-fed without Cry1C, and therefore cannot persist and colonize the gut [37, and 59].

### Galleria mellonella *survival and gut colonization of* B. cereus *following ingestion*

**Figure 1.**
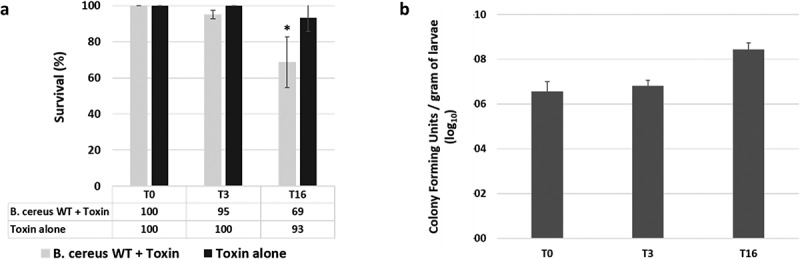
**Survival and gut colonization level of *Galleria mellonella* after oral infection with vegetative *Bacillus cereus* ATCC 14,579 (WT)**. A) *G. mellonella* survival was recorded immediately (T0), 3 hours (T3) and 16 hours (T16) after force-feeding with *B. cereus* WT + Cry1C toxin or toxin alone. B) *B. cereus* WT persistence inside the whole *G. mellonella* gut was measured at T0, T3, and T16. Error bars represent standard deviations of three independent experiments. 40 larvae were force-fed per experiment. For CFU counts a total of 18 larvae (3 x 6 larvae) were analyzed per time point. Statistical differences are annotated. The student t-test was performed using RStudio software (n = 3, *p* < 0.05)

Therefore, we measured *G. mellonella* survival after force-feeding with a suspension of *B. cereus* and Cry1C toxin. Control was performed with Cry1C toxin alone, and data were recorded immediately (T0), 3 hours (T3), and 16 hours (T16) after force-feeding (Figure 1a). A survival rate of 69% of *G. mellonella* larvae was observed T16 after *B. cereus* and toxin ingestion, which is statistically different from the control batch fed with Cry1C toxin only (93% of survival). No difference is recorded at T0 and T3, despite a few dead larvae (95% of survival). In addition, survival at 24 hours was 95% for toxin alone and 49% for the association of bacteria and toxin. To determine bacterial persistence inside the gut in living *G. mellonella* larva, we chose the time points planned to be used for micro-dissection (3 hours and 16 hours post infection), and for which less than 30% mortality was observed. We assessed the number of *B. cereus* recovered in the whole larva (Figure 1b). The amount of *B. cereus* increased throughout the infection time, with a mean value of 3.75 × 10^6^ CFUs (Colony Forming Units) *per* gram of larvae (about 5 larvae for one gram) at T0, to 6.51 × 10^6^ CFUs *per* gram of larvae at T3 and 2.77 × 10^8^ CFUs *per* gram of larvae at T16. Despite an increase of two log value at T16 compared to T0 and T3, this difference was not statistically different due to variation among replicates. Based on these results, we were confident to find *B. cereus* in alive insect larvae at both T3 and T16 after force-feeding.

### *LCM of* B. cereus *in* G. mellonella *midgut for qPCR analysis*

**Figure 2.**
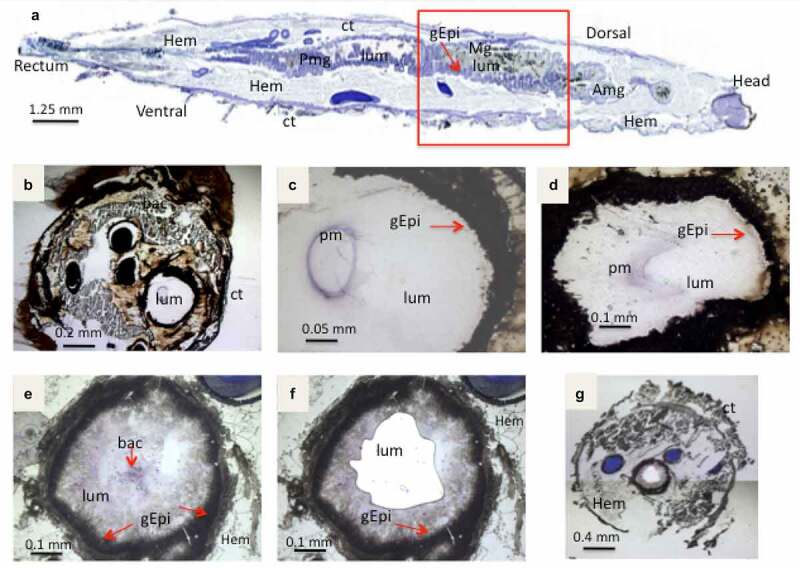
**Light microscope observation of stained cryo-sections of germ free *G. mellonella* larvae, Sections in (A,B,C,D) are without *B. cereus*, while E,F,G shows the UV-micro-dissection of bacteria in the Larval lumen 3 hours post force-feeding with *B. cereus* associated with toxin**. (a) a sagittal20 µm thick-section of a whole non-infected larvae. The area inside the red square indicates the part from which cross-sections (16 µm thick) were sampled for laser-capture microdissection. (b, c) Cross-sections from a negative control (PBS force-fed) larvae (d) section from larva T3 hours post force-feeding with Cry1C toxin alone (B,C,D are captured with lens: 10×). The figures E, F and G present cross-sections before (e) and (f) after laser-capture microdissection with UV-cutting (F, lens 10×; G, lens 2×). (lum: Lumen; Hem: Hemocoel; Ct: Cuticle; Mg: Midgut; Amg: Anterior midgut; Pmg: Posterior midgut; gEpi: Gut Epethlium (enterocytes); pm: Peritrophic matrix; bac: Bacteria

To compare the expression of *B. cereus* genes during *G. mellonella* midgut infection, two time points were selected. Three hours (T3), designed as control to allow bacterial adaptation in the intestinal environment (supposed to be in the lumen before adhesion to the gut epithelium), as well as sixteen hours (T16), when the bacteria should be colonizing the intestinal surface. This observation was based on non-published histology analysis with the *B. thuringiensis* 407cry- strain, which, like *B. cereus* 14579, does not carry Cry toxin genes. To decide which part of the intestine to analyze, infected frozen *G. mellonella* larvae were cut in four equal cross-sections, and the number of *B. cereus* CFUs inside each section was determined. At T16, bacteria were recovered in each larval section; the majority was in the second section corresponding to the upper part of the midgut (Figure 2a), which we then used for the sampling. To perform LCM, we cut the area containing the bacteria with the UV laser and then glued it to the bottom of the macro cap.

### Histology and infection progress

First, larvae from the negative control, force-fed with PBS buffer, were analyzed under the microscope following cresyl violet staining (Figure 2b & 2c). The intestinal surface and the peritrophic matrix (violet ring in the lumen) were visualized and looked intact. Larvae treated with Cry1C only showed a modified peritrophic matrix at T3 post ingestion (Figure 2d), suggesting that Cry1C might structurally damage this barrier. The UV-cutting laser was used to collect *B.* *cereus*samples inside *G.* *mellonella* lumen (Figure 2e, 2f and 2g). UV dissected sections shown on figure 2f and g, indicate that a whole area inside the intestinal lumen has been removed.

**Figure 3.**
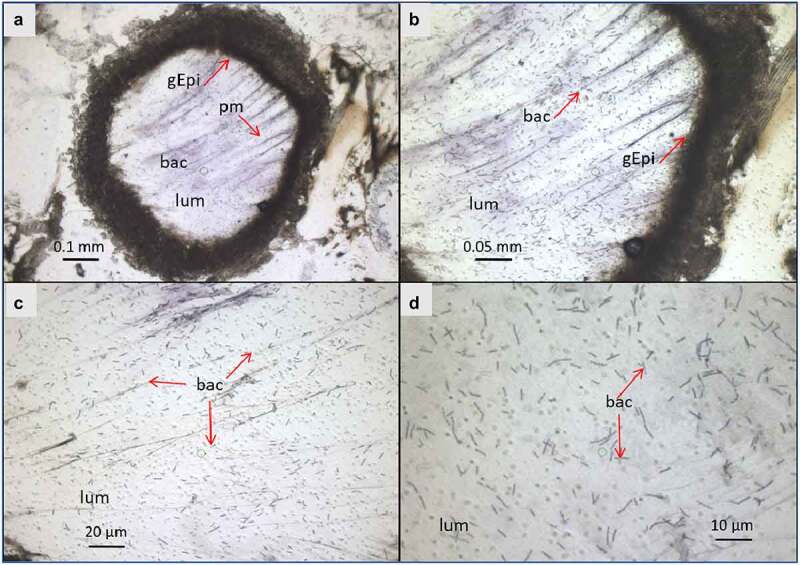
**Light microscope observation of G. mellonella larvae infected with B. cereus associated with Cry1C toxin 3 hours after force-feeding. Cross–section (16 µm) with focus on the midgut lumen, and bacterial localization and morphology** . The sections were stained with cresyl violet and dehydrated with ethanol and xylene (a). The majority of the bacteria were found in the midgut lumen and the peritropfic matrix has lost its structure. (A) lens 4×; (b) lens 10×; (c) 20× lens, and (d) 40× lens. lum: intestinal lumen; bac: bacteria; gEpi: Gut Epithelium; pm: Peritrophic matrix

**Figure 4.**
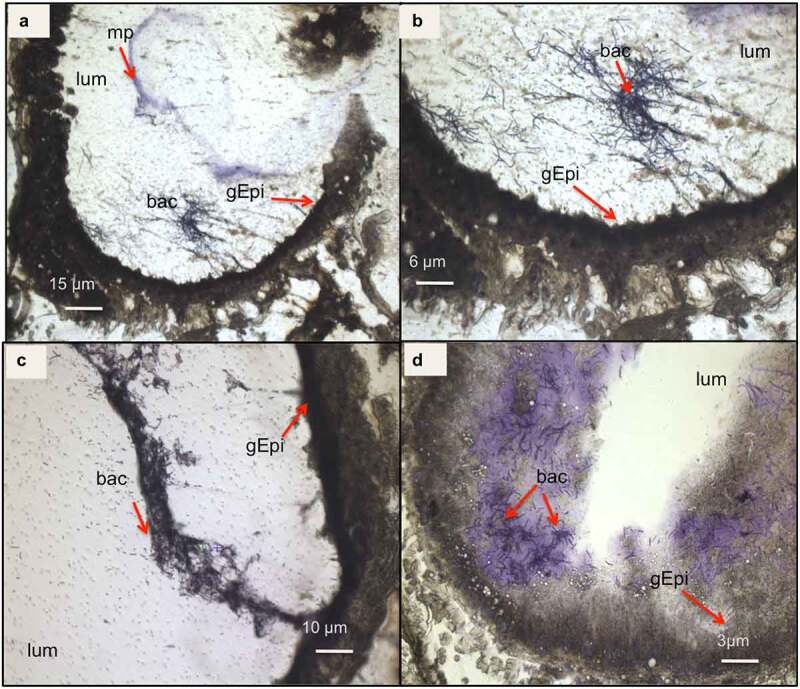
**Light microscope observation of *G. mellonella* larvae infected with *B. cereus* associated with Cry1C toxin 16 hours after force-feeding**. Cross–sections (16 µm) (cresyl violet staining and ethanol and xylene dehydration) with focus on the intestine (midgut). The bacteria appear colored as dark or brighter violet. Different magnifications are shown: (a) 4×; (b)10×; (c) 20×, and (d) 40×. (lum: intestinal lumen; bac: bacteria; gEpi: Gut Epithelium; pm: Peritrophic matrix

At T3, the bacteria were widespread in the center of the lumen (Figures 2.E and 3)

while being, at T16, either closer to the gut epithelial or embedded in detached epithelial cells in the lumen (Figure 4). At both T3 and T16, *B. cereus* crossed the peritrophic matrix, which could not be properly identified anymore, probably due to the Cry1C toxin and/or the bacteria associated synergy action [37]. The gut epithelium kept its integrity at T3, while some enterocyte damage was observed at T16, which could enable the bacteria to enter the hemocoel. Differences in bacterial shape and localization in the lumen were noticed when comparing the two time points (Figures 3 and 4). While *B. cereus* was widespread and more individualized in the gut lumen at the early stage of infection (T3), they tended to concentrate closer to the epithelium at T16, forming aggregates like a potential biofilm. These observations suggest physiological and phenotypical adaptation of *B. cereus* to this step of infection. Hence, as expected, the bacteria increase the intestinal cell deterioration, which later results in larval disease or mortality due to bacterial proliferation in the hemocoel.

### RNA extraction and cDNA production

**Figure 5.**
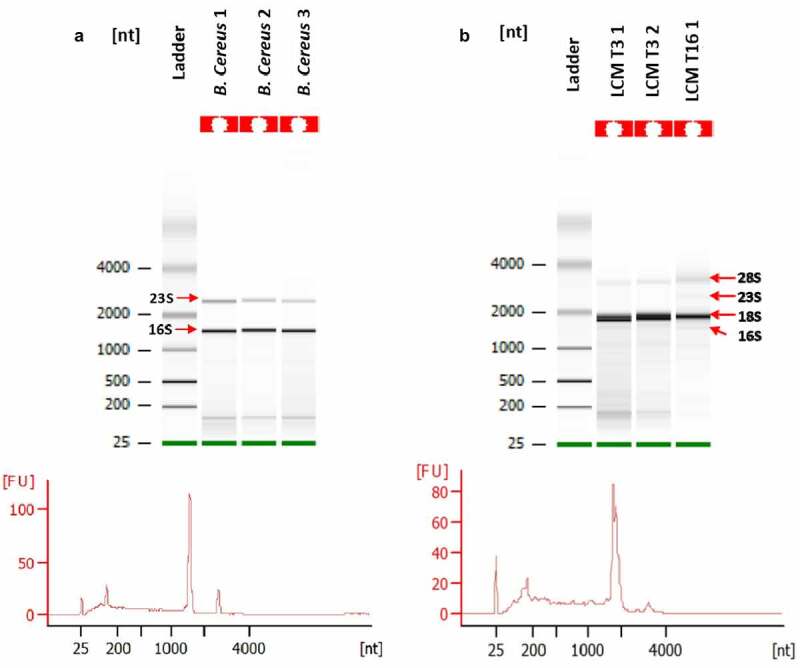
**Quality assessment or total RNA (totRNA) samples (Agilent Bioanalyzer**). A: totRNA quality of *B.cereus*cultures; B: totRNA quality of *G.mellonella* gut infected with B. cereus associated with Cry1C toxin obtained after Laser-Capture Microdissection (LCM) at 3 hours (T3) post force-feeding and 16 hours (T16) post force-feeding. 16S and 23S indicate bacterial ribosomal RNA. 18S and 28S indicate eukaryotic ribosomal RNA

After gathering the collected samples, the quantity and quality of total RNA were assessed. We compared the bioanalyzer results of total RNA (totRNA) collected with LCM to totRNA extracted from a *B. cereus* planktonic culture stopped at OD_600nm_ = 1 (Figure 5a and b). Low amount of totRNA was recovered and more inconveniently, the large majority of totRNA was eukaryotic, even though the dissected areas were thoroughly designed to target bacteria and to avoid visible epithelium cells of insect’s gut.

**Figure 6.**
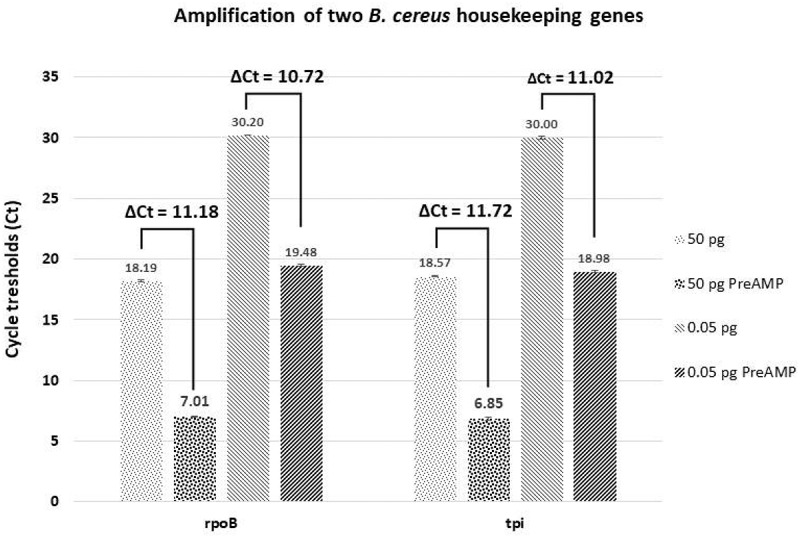
**Gene amplification test using two different quantities of cDNA obtained from B. cereus grown in vitro**. The cycle threshold (Ct) values are obtained by qPCR for each condition tested are indicated. Difference of Cts (ΔCt) between the control and the pre-amplified (PreAMP) cDNA for each gene and condition are indicated as well. 50 or 0.05 pg: the quantity of cDNA tested in control samples (without pre-amplification); 50 or 0.05 pg PreAMP: the quantity of cDNA tested from PreAMP samples. Each gene exhibits about an 11 Ct gain when preamplified for each quantity tested. Results were obtained from three independent LB cultures and each qPCR analysis was performed with three technical replicates per sample

To overcome this very low input of *B. cereus* total RNA and the presence of eukaryotic RNA, only specific genes were targeted in qPCR after a pre-amplification (PreAmp) step. Target-specific PreAmp is usually performed by multiplex PCR using a predefined primer pool [65]. Thereby, we were able to analyze several target genes from micro-dissected samples. However, the main challenge of the amplification protocol is to preserve the relative transcript levels [39]. Today, several kits are commercialized with very reproducible results. In this study, to validate the PreAmp reliability, two *B. cereus* housekeeping genes, *rpoB* and *tpi*, were chosen. The cDNA analyzed was obtained from mRNA extracted from *in vitro* cultured *B. cereus*. For both genes and for two cDNA quantities templates, a Ct gain around 11 Ct was obtained. Indeed, with 50 pg and 0.05 pg cDNA the Ct gain was 11.18 and 10.71 Ct for *rpoB* respectively and for *tpi* 11.72 Ct gain was found for 50 pg cDNA and 11.02 Ct for 0.05 pg (Figure 6). The efficiency and reliability of this kit was therefore established in our conditions with both low-input (50 pg), or very low-input (0.05 pg) of cDNA.

### *qPCR expression of iron homeostasis genes from* in vitro *collected bacteria*

Before analyzing the gene expression *in vivo,* we set up a control measure in order to know how some of the chosen iron homeostasis genes were expressed *in vitro*, under iron restricted condition. We compared (ΔΔCt) fold change between bacteria collected at mid-log phase growth phase from LB medium and from moderate iron depleted medium (LB + Dip 0.2 mM). The results indicated in Figure S1, show that *feuA, entA, fatB* were upregulated about 2 fold, *asb* 1.4 fold and *ilsA* was 4 fold more upregulated in LB-Dip, compared to LB alone. This indicates that the studied genes, as expected, were more highly expressed in the iron-restricted condition than in iron-rich condition.

### *qPCR expression of iron homeostasis genes from* in vivo *collected bacteria*

**Figure 7.**
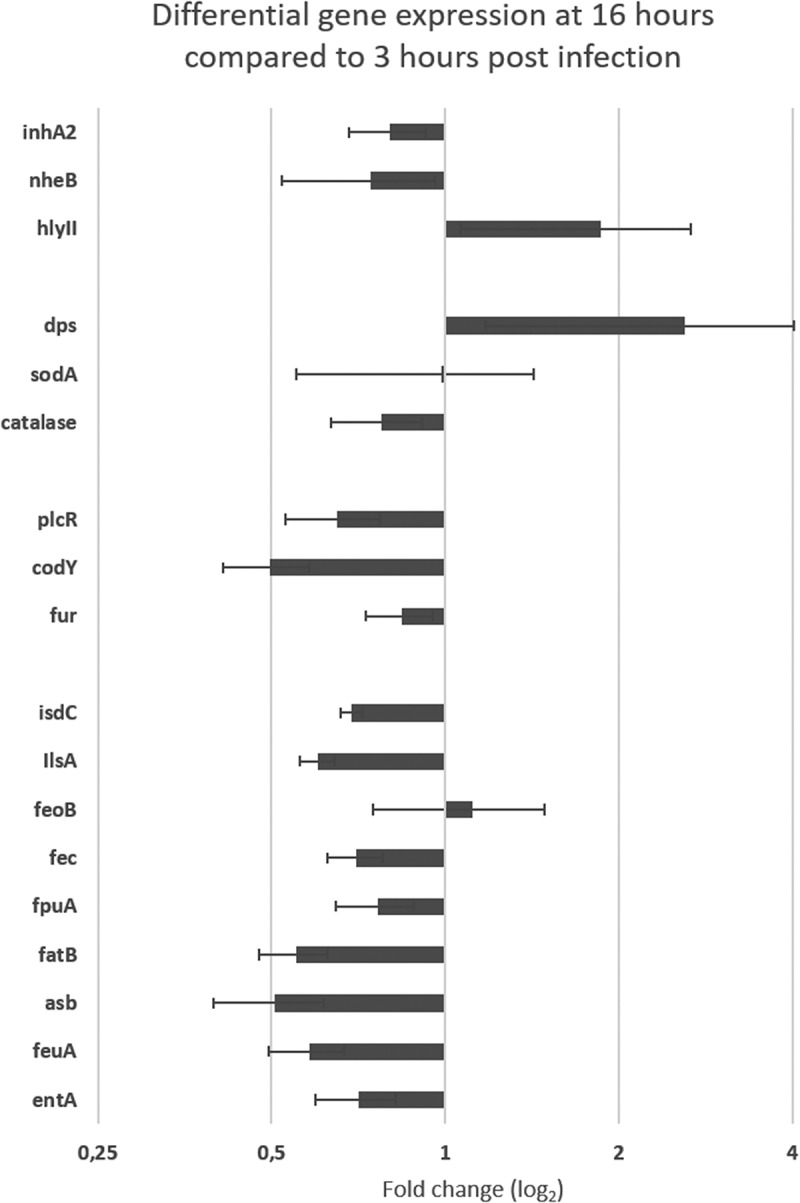
**Differential (**ΔΔCt) **gene expression of *B.* *cereus* ATCC 14,579 associated with Cry1C toxin** collected at late infection stage (16 hours) compared to early stage (3 hours) post force-feeding in the midgut of germ-free *G.* *mellonella*. mRNA extractions were from LCM sections obtained at the two time points. Data are normalized using 3 housekeeping genes (rpoB, tpi, and purH). Error bars indicate the standard deviation of three biological replicates. Secreted virulence factors (inha 2 (metalloprotease), nheB (Non hemolytic enterotoxin); hlyII (hemolysin II); Oxidative stress factors : sodA (superoxidasedismutase); catalase (catalase); dps (ferrtiin–like iron binding protein); Regulators : plcR (virulence regulon transcriptional activator); fur (Ferric uptake regulator (often repressor);codY (Transcriptional repressor). Iron homeostasis factors: isdC (Heme associated transport protein), ilsA (iron regulated leucine–rich protein), feoB (Ferrous iron transport protein); fec (Iron III) dicitrate ABC transporter); fpuA (petrobactin siderophore binding protein); fatB (petrobactin siderophore binding protein); asb (gene from the petrobactin siderophore synthesis locus); feuA (bacillibactin siderophore binding protein); entA (gene from bacillibactin siderophore synthesis locus)

To survey whether genes involved in *B. cereus* iron homeostasis play a role during gut colonization, we targeted genes that could help to understand the environmental context and physiological stress triggered in *B. cereus* at both chosen times of host colonization (Table 1). The amplification method we performed was thus used to measure *B. cereus* gene expression at late stage compared to early stage of *G. mellonella* midgut infection (Figure 7). The fold change of gene expression at T16 compared to T3 was normalized using three housekeeping genes: *rpoB, tpi,* and *purH*. Genes involved in iron homeostasis and controlled or not by Fur (*fur* appears slightly down-regulated: 0.84 fold change) are down regulated, except *feoB*, which corresponds to a protein involved in the ferrous (Fe^2+^) iron transport (1.12 -fold change). Two other genes were overexpressed at T16, *hlyII* and *dps*, with expression ratios of 1.86 and 2.60 (fold change) higher, respectively. Interestingly, genes involved in oxidative stress had different behavior. While *dps* was up-regulated (2.60-fold change), the superoxide dismutase *sodA* had a level of expression (0.99 -fold change) similar to the early infection stage, and finally the catalase was down-regulated (0.77 -fold change). Also, the genes encoding the virulence factors *nheB* (non-hemolytic enterotoxin) and *inhA2* (metalloprotease), both controlled by the transcriptional activator PlcR, were slightly down regulated (0.74 and 0.80-fold change, respectively). Finally, *plcR* itself was down regulated (0.65-fold change), as well as the global transcriptional regulator *codY* (0.50-fold change).

### G. mellonella *iron homeostasis genes responses*

**Figure 8.**
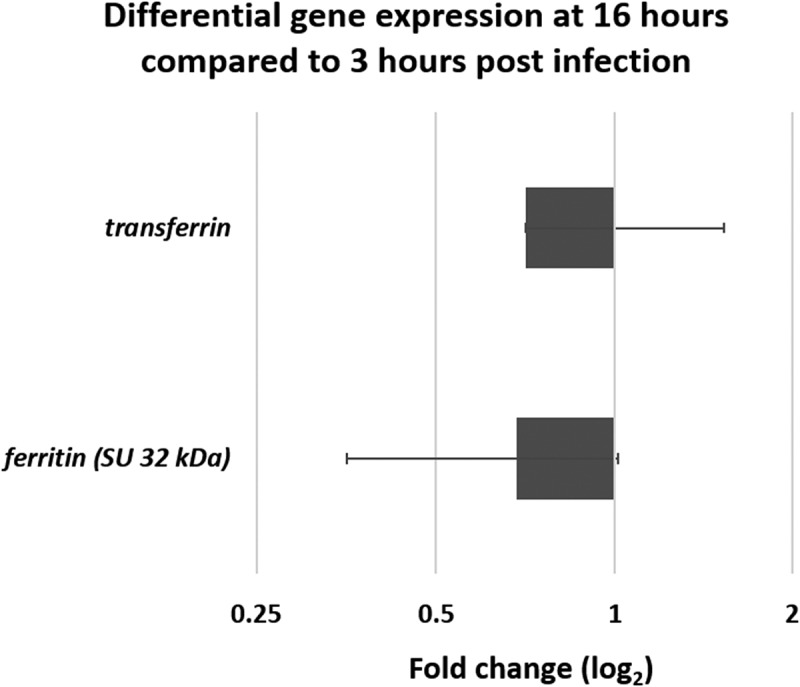
**Differential gene expression (**ΔΔCt) **of two iron related *G. mellonella* genes**. Genes encoding ferritin and transferrin obtained from mRNA at late infection stage (16 hours) compared to early stage (3 hours) in germ-free *G. mellonella* midgut infected with *B. cereus* associated with Cry1C toxin. Error bars indicate the standard deviation of three biological replicates

To complete the host-pathogen study, an analysis of the expression of *G. mellonella* genes identified as important in dealing with iron homeostasis was also conducted. These are the gene encoding the 32 kDa subunit of ferritin, an iron storage and transport protein, and the gene encoding for transferrin, also involved with iron transport protein and considered involved in nutrient immunity. The results (Figure 8) reveal that transcripts of both of these genes are recovered in the lumen of *G. mellonella* gut. Ferritin is slightly down-regulated at T16 compared to T3 (0.71) while transferrin is slightly upregulated with a 1.68 fold change. The raw Ct values are reported in Table 2S.

### Discussion

The main goal of this study was to achieve an *in vivo* analysis that could provide additional insight of *B. cereus* adaptation during intestinal infection, through the help of an insect model. To achieve this, a novel and targeted approach was designed to assess whether well-known genes involved in iron homeostasis, virulence, and adaptation factors were differentially expressed during the intestinal infection process. Indeed, little is known about iron status of entero-pathogenic Gram-positive bacteria in the gut environment: how bacteria adapt in the intestinal lumen, and which bacterial factors are activated when the host cells are damaged. By using the LCM approach, we were able to study *in vivo* the adaptation of *B. cereus* during the *G. mellonella* gut infection, for the first time.

An early stage of infection at 3 hours post-ingestion was chosen, compared to a later stage, at 16 hours. The focus was on genes involved in iron homeostasis; however, other genes known as indicators for specific bacterial metabolic activities were also chosen. Through this approach, an assessment could be made on the bacterial stress in the host environment. Finally, the expression of a few genes encoding virulence factors and two *G. mellonella* genes involved in host iron homeostasis were also analyzed.

The use of LCM seemed appropriate given the increased interest for this technique to decipher host–pathogen interactions, with respect to microbial aspects [41,42]. Meanwhile, in those papers, the targeted genes were focusing on host responses or identification of microbiota species. To our knowledge, none of these studies were specifically focusing on pathogen gene expression at different time points of gut colonization. Therefore, our study provides evidence for an additional use of LCM for targeted *in situ* gene expression of an extracellular pathogen.

Numerous kits allowing amplification of low or very-low input RNA from small samples, as well as single cell sequencing methods, have been used and compared to perform RNA sequencing [66,67]. The lack of poly-A tails in prokaryotic RNAs makes it impossible to use an amplification kit based on oligo-dT priming, and therefore reduces the number of available kits for bacteria sampling. Some kits proposing poly-A tailing before amplification have been used in previous studies [52], and adaptation with a poly-A tailing kit before choosing another amplification kit was considered. This was not chosen, as we wanted to avoid an additional step that could bias the analysis. The ratio between total RNA amounts of prokaryotic cells compared to eukaryotic cell signifies an issue. Some techniques, to enrich bacterial mRNA, or to perform RNA sequencing for both bacteria and host cells during infection, have been proposed in that regard [68,69]. Performing RNA sequencing as output of the LCM sampling was too uncertain, since the amount of eukaryotic RNA was much higher compared to the prokaryotic one, with a ratio around 1/1000 in our experiments, as measured by Bioanalyser. These observations indicate the presence of large amount of *G. mellonella* RNA in the lumen outside the epithelial surface, likely caused by cell apoptosis, gut cell turnover, and cell lysis due to pore forming toxins and proteases [70,71]. Also, these factors, as well as an increased stability or slower turnover of eukaryotic mRNA, compared to the bacterial one, might also be a reason. The qPCR coupled with target-specific pre-amplification used in our study is recognized as a powerful tool to analyze differences in targeted gene expressions [72] starting from a low amount of sample [65]. Therefore, the basis for our analysis should be reliable.

In this study, we hypothesized that the modulation of *B. cereus* gene expression related to iron homeostasis helps the bacteria to settle during host gut infection. For most of the targeted genes, the expression was down regulated at 16 hours post-infection, compared to the chosen control stage 3 hours post-infection. These results suggest that the apparent lower iron availability at T3 impose higher expression of *B. cereus* iron-related genes, until the available iron amount in the gut environment is increased, as shown by a lower expression at T16 of iron homeostasis-related genes. Genes involved in iron homeostasis, entA, *feuA, fatB, fec, feoB, IlsA, IsdC*, (all controlled by Fur), and two Fur-independent genes (*fpuA, asb*) were thus all down regulated at the later stage of intestinal infection (T16), except *feoB*, which did not change. It has been previously shown that *in vitro*, Fur-controlled genes involved in iron uptake are down-regulated in iron replete conditions, except the *asb* operon, which encodes the siderophore petrobactin [24].

We also performed a control experiment *in vitro* to be compared with the fold-change values we obtained from the *in vivo* collected bacteria. Hence, the level of targeted *B. cereus* gene expression involved in iron homeostasis, from cultures in iron chelated medium, was compared to expression in iron rich Luria-Bertani medium (Figure S1, Table S1 and S3). The results indicated an overall higher expression in iron-chelated medium. Interestingly, the level of differential expression recorded in the *in vitro* control and in our *in vivo* study has comparable values (around twofold change). Therefore, these results may underline that iron homeostasis is tightly regulated and that many genes involved in iron uptake and export are continuously expressed at a certain level, in the host gut.

However, the expression of genes does not directly indicate a role in adaptation or virulence. Meanwhile, it was previously shown in *G. mellonella* mortality tests that the mutant of both *entA* (first gene of the operon expressing the siderophore bacillibactin synthesis) and *ilsA* (a ferritin and hemoglobin surface binding protein) conferred less mortality than the Bc ATCC14579 parental strain [15,19] following hemocoel infection. In the present study, both *entA* and *ilsA* were expressed in the gut. Hence, we suggest that both bacillibactin and IlsA might take part in bacterial adaptation in the gut and not only after reaching the hemocoel, as was previously suggested for IlsA based on transcriptional fusion between the *ilsA* promoter and *gfp* or *lacZ* plasmid born reporter constructions. Indeed, fluorescence or β-galactosidase activity was only observed in the hemocoel and not in the gut [15,38]; this discrepancy might be due to the higher sensitivity of qPCR analysis. Indeed, only a minor gene expression can result in PCR amplification while the transcriptional promoter reporter fusion requires a certain level of promoter activity and a good stability of the reporter molecules.

In the following discussion, we aim at highlighting the function of other studied factors and associate their expression with host adaptation or host damage. In late infection stage, the upregulation of *feoB* appears not different from the early stage (1.4 and 1.2 vs. 0.7 for the third). The membrane protein FeoB was identified as a G protein with GTPase activity [73], allowing import of ferrous iron in bacteria [74]. These results suggest that *B. cereus* can use this system in iron uptake supposing that ferrous iron is present in the insect gut environment.

To prevent damages caused by reactive oxygen species (ROS) released in the environment, bacteria have to use strategies for DNA repair, lipid membranes, or proteins protection [75]. The bacterioferritin Dps is involved in iron storage and protects bacteria from oxidative stress *in vivo* [76]. In our conditions, this gene harbors the largest positive differential expression at the late stage of infection (T16). It indicates that inside the gut, the oxidative stress is increasing, probably due to host cell degradation, hence releasing molecules of ferrous iron in the environment, which reacts with oxygen to produce reactive oxygen species (ROS) by Fenton reaction. Two other genes were targeted as indicators for oxidative stress inside *G. mellonella* gut along the infection process: the superoxide dismutase *sodA* and a catalase gene. It is known that both of these factors are protecting bacteria from ROS, especially superoxide radicals and H_2_O_2_ [77], and could be up or down regulated in Gram-positive bacteria depending on the metal released in the environment [78]. Here, while the *catalase* gene was down-regulated, *sodA* varied among replicates (1.1, 1.3, and 0.5 – fold change), revealing an expression of almost similar level at the two time points. Therefore, our results do not clearly indicate an increased oxidative stress at late compared to early colonization, but the increased expression of *dps* tells that at T16 probably more iron is available and storage is needed to prevent damage in *B. cereus*.

*In vivo* assays in *G. mellonella* following injection of *B. thuringiensis* into the hemolymph were previously performed to study the level of expression of the Hemolysin II (*hlyII*) and its importance in virulence [71,79]. To our knowledge, *hlyII* expression has not been tested during oral infection in *G. mellonella*. In our study, *hlyII* is overexpressed in late infection stage compared to the beginning of infection. This gene is under control of its own regulator HlyIIR, and the major iron regulator Fur [22,80]. *HlyII* expression is induced in the hemocoel with low iron and glucose availability [71,81] allowing the bacteria to access nutrients and promoting growth by degrading host phagocytes and erythrocytes [82]. Even though *fur* is slightly down regulated at T16, the bacteria are still expressing *hlyII* when they are close to the epithelium cells. The resulting enterocyte cell lysis may then provide nutrients for the bacteria. The pore-forming activity of HlyII on *G. mellonella* gut cells remains to be studied, but one might speculate that the increased production of HlyII resulting in gut cell damage would release ferrous iron, which could be imported by the Feo system, as known for Gram negative enterobacteria [16].

It was earlier published that the metalloprotease InhA2 has a role in *B. thuringiensis* virulence following oral infection of *G. mellonella*, most likely to help to cross the gut barrier [83]. Our results show that expression of *inhA2* is slightly decreased at late infection stage compared to early infection stage (fold change: 0.8). These results indicate that the epithelium degradation probably started early during infection. Interestingly, the gene encoding its transcriptional activator, PlcR, is also down regulated at T16. PlcR is known to be a sensor for certain environmental factors expressed *in vitro* at the early stationary phase [84]. The expression of PlcR regulated genes during *G. mellonella* gut infection was expected since the PlcR regulon is important for full virulence of *B. thuringiensis* and *B. cereus*, following oral infection in *G. mellonella* [37]. Furthermore, studies focusing on the activation of the PlcR-PapR quorum sensor system in another insect indicate that PlcR-PapR dependent genes should be activated *in vivo* [85].

Although this study was designed to decipher variations in bacterial gene expression, we also looked at host ferritin and transferrin gene transcripts. These proteins have major roles in host iron homeostasis, but also in response to infections [64]. Ferritin serves in insects as both storage and transport for thousands of Fe^3+^ iron and transferrin for transport and delivery of Fe^2+^ to the host cell [54]. These molecules are also considered as host immune response effectors and are therefore expressed as soon as the host encounters a pathogen. In our analysis, we can simply state that we observe expression of the genes encoding transferrin and ferritin at both time points in the presence of the pathogen. Hence, further work is required, especially with non-infected larvae to enlarge the knowledge on host responses modulation with and without intestinal pathogens and intestinal microbiota.

In conclusion, this study set up a model giving a new insight to *B. cereus* colonization and *in vivo* and *in situ* adaptation during gut infection. We demonstrate that this challenging LCM approach is feasible to investigate bacterial gene expression, even with a low amount of bacterial RNA collected. Genes involved in iron homeostasis could play a key role for bacterial colonization and development inside the gut, since these genes are expressed at both early and later gut colonization. However, the relative roles of the encoded factors remain to be studied for several of them. Furthermore, this study is opening perspectives for larger transcriptome analyses for both bacteria and the host in order to get a host pathogen crosstalk analysis. *G. mellonella* is largely used as an alternative to mammalian models of infection at the innate cellular and humoral defense level [34]. We show here that it is also useful for in-depth study of pathogenic gut bacteria. Moreover, the possibility to use germ-free *G. mellonella* larvae makes this infection model even more attractive and could reduce the number of experimental mammals. Only few studies using mammals to explore *B. cereus* virulence and gene expression or toxin detection are available [9,86,87]; whether the observation here performed in *G. mellonella* is reliable for *B. cereus* mammal intestinal infection remains open. Nevertheless, this study shows the expression of the enterotoxin component NheB in a gut environment and it would be interesting to analyze the expression of other *B.cereus* enterotoxins as well.

## Supplementary Material

Supplemental Material
