# Supplementary material for: Laser capture microdissection to study Bacillus cereus iron homeostasis gene expression during Galleria mellonella in vivo gut colonization
Source: Virulence. 2021 Aug 10;12(1):2104–21. doi: 10.1080/21505594.2021.1959790 (PMC8366545; doi:10.1080/21505594.2021.1959790)
Supplement: Supplemental Material [file KVIR_A_1959790_SM2882.zip › supp.pdf]

Supplementary data:

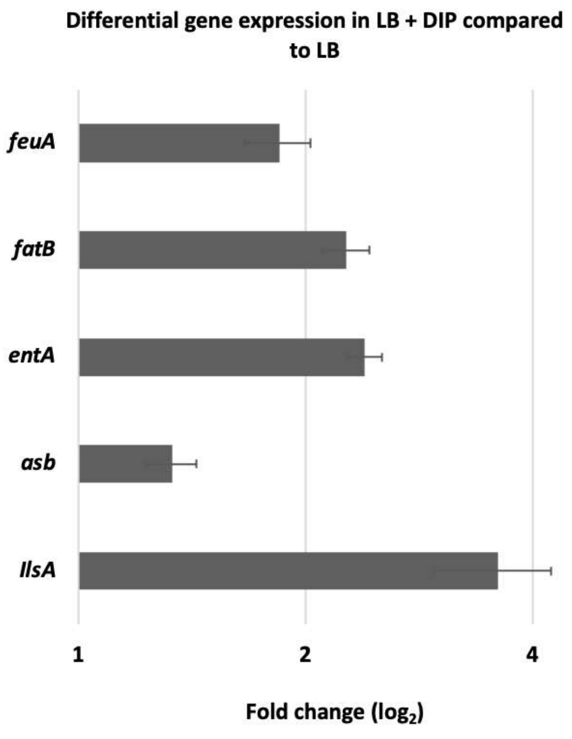

Figure S1: Differential *in vitro* gene expression of *B. cereus* iron homeostasis related genes. The expression analysis ΔΔ Ct shows expression in an iron chelated growth medium (LB+DIP 0.2mM) compared to an iron rich LB medium. The bacterial cultures are harvested at a similar growth stage (mid log phase) for both cultures. All genes are overexpressed in iron chelated conditions. The expression was normalized with 2 reference genes (*rpoB, tpi*). The error bars represent the mean variation from 3 repetitions (three independent cultures for each medium with 3 technical replicates per culture).

Table S1: Raw Cycle threshold (Ct) values from qPCR analysis
Bacillus cereus studied genes expressed in *G. mellonella* gut (obtained from preamplified cDNA).

Gene name	Mean Ct 3 Hours	Standard deviation 3 Hours	Mean Ct 16 Hours	Standard deviation 16 Hours
aAsb	18.06	0.54	18.36	2.30
calY	16.33	0.49	16.29	2.44
catalase1	16.18	0.16	15.89	1.98
codY	13.93	1.80	14.29	0.51
dDps	15.67	0.31	13.79	0.92
Enta	26.97	0.99	26.81	3.07
fatB	17.40	0.40	17.56	2.04
fFec	16.75	0.35	16.59	2.47
feoB	14.88	1.05	14.11	0.73
feuA	17.26	0.48	17.35	2.32
fpuA	18.10	0.42	17.83	2.30
fur	13.20	2.25	12.79	0.35
hlyII	20.91	1.18	19.42	1.05
ilsA	17.00	0.04	17.02	2.09
inhA2	16.84	0.32	16.50	2.14

isdC	16.81	0.35	16.68	2.59
ivi6	16.79	0.23	16.47	1.93
narg	17.51	0.31	17.49	2.04
nheB	16.76	0.49	16.53	1.36
plcR	16.35	0.35	16.29	2.00
purH*	17.25	0.19	16.81	2.20
rpoB*	13.39	3.89	12.53	0.88
sodA	16.33	0.27	15.79	1.39
tpi*	13.96	2.86	12.77	1.23

* refers to reference genes

The mean values are obtained from 3 technical retriplicates from three biological rReplicates. Stardard deviations are obtained using Exell software.

Table 2S: **Raw Ct values from qPCR -(obtained from preamplified cDNA) analysis of *G. mellonella* genes -expressed in the gut during infection with *B. cereus***

Sample	Target gene	Average	Standard deviation
**3 hours**	***EF 1***	7.93	0.85
	Gm Ferr32	8.18	0.41
	transferrin	12.12	1.02
**16 hours**	***EF 1***	8.39	1.36
	Gm Ferr32	9.31	1.96
	transferrin	14.39	4.01

Table S3: Raw Ct (Cycle threshold) values from -qPCR -analysis . *Bacillus cereus* studied genes expressed *in vitro* in LB medium or in iron chelated LB+Dip (0.2 mM) medium from non preamplified cDNA. The total RNA (including mRNA) was extracted from both cultures at their similar mid log growth phase.

Sample name	Target Name	Mean adjusted Equivalent Ct
**LB** **	*ilsA*	19.69
	asb	21.59
	entA	23.91
	fatB	20.15
	feuA	20.39
	*purH**	19.99
	*rpoB**	17.53
	*tpi**	18.97
**LB + DIP** **	*ilsA*	17.93
	asb	21.26
	entA	22.73
	fatB	19.05
	feuA	19.59
	*purH**	20.00
	*rpoB**	17.80
	*tpi**	18.93

* refers to reference genes

(** the qPCR is performed with an amount of cDNA obtained from 1 ng of extracted total RNA).

Ct values are obtained from three independent repetitions (cultures) and from three technical replicates.
